# Work-Related Musculoskeletal Disorders Among Radiology and Ultrasound Technologists During Ramadan in Madinah, Saudi Arabia: A Cross-Sectional Study

**DOI:** 10.3390/healthcare14172718

**Published:** 2026-08-26

**Authors:** Abdulkareem Algahtani, Jory Alluhaibi, Monaya Altamimi, Nada Almutairi, Manar Alrehaili, Dalia Alsahli, Bashair Alhummayani, Mohammed Asiri, Abdulhadi Alkulib, Sultan Alshoabi, Awadia Gareeballah, Awatif M. Omer, Moawia Gameraddin, Reem Elkady, Kinan Mokbel

**Affiliations:** 1 Department of Diagnostic Radiology, College of Applied Medical Sciences, Taibah University, Madinah 42353, Saudi Arabia; 2 Department of Radiological Sciences, College of Applied Medical Sciences, Taif University, Taif 21944, Saudi Arabia; 3 Medical Imaging Department, King Faisal Medical City, Abha 62523, Saudi Arabia; 4 Department of Health and Care Professions, Faculty of Health and Life Sciences, University of Exeter, Exeter EX1 2LU, UK

**Keywords:** Ramadan, radiology technologists, radiographers, ultrasound technologists, sonographers, sonography, radiography, musculoskeletal symptoms, work-related musculoskeletal disorders, WRMSD, occupational health

## Abstract

**Background:** Radiology and ultrasound technologists are exposed to sustained static postures, repetitive movements, equipment handling, and patient-positioning tasks that increase the risk of work-related musculoskeletal disorders (WRMSDs). During Ramadan, increased healthcare demand associated with Umrah may further increase occupational workload in Madinah. **Objective:** To estimate the 7-day prevalence of WRMSDs and identify occupational factors associated with pain intensity among radiology and ultrasound technologists working during Ramadan in Madinah. **Methods:** A cross-sectional online survey was conducted among 237 radiology and ultrasound technologists working in Madinah during Ramadan 1447/2026. Participants were recruited using online convenience and snowball sampling through professional networks and social-media platforms. The survey included the Nordic Musculoskeletal Questionnaire (NMQ), a 0–10 Numeric Pain Rating Scale (NPRS), and demographic and occupational variables. Bivariate analyses and multivariable linear regression with robust standard errors were used to examine factors associated with pain intensity. **Results:** Overall, 89.5% of participants reported WRMSDs during the preceding 7 days. The lower back (56.5%) and neck (40.5%) were the most affected regions. Mean pain intensity was 5.55 ± 2.28. Higher pain intensity was associated, after adjustment for the variables included in the model, with older age, working as an ultrasound technologist, managing 11–20 or more than 30 patients per shift, and frequent night-shifts, with the strongest association observed among participants working at least three-night-shifts per week (B = 3.27, 95% CI 2.43–4.10). BMI, sex, years of experience and shift duration were not independently associated with pain intensity after adjustment. **Conclusions:** WRMSDs were highly prevalent among radiology and ultrasound technologists working during Ramadan in Madinah. Night-shift frequency and very high patient workload showed the largest occupational associations with pain intensity. These findings identify workload distribution and night-shift scheduling as potential areas for occupational health evaluation during periods of increased healthcare demand. Because of the cross-sectional design, the findings should be interpreted as associations rather than causal effects.

## 1. Introduction

Work-related musculoskeletal disorders (WRMSDs) are a major occupational health concern among healthcare workers and a contributor to occupational disability. Radiology technologists are exposed to patient transfers, positioning manoeuvres, repetitive equipment handling and prolonged static postures. Ultrasound technologists, or sonographers, face additional ergonomic risks related to sustained shoulder abduction, repetitive wrist movements, prolonged transducer grip and continuous transducer pressure during scanning [1,2,3].

Fasting from dawn to sunset during Ramadan is associated with changes in meal timing, sleep timing, circadian rhythm and physical performance [4,5,6,7,8]. Ramadan has been associated with changes in sleep architecture, delayed sleep timing, reduced total sleep time and altered daytime functioning [4,6]. These changes may reduce recovery from occupational loading and increase susceptibility to musculoskeletal symptoms, particularly when combined with shift work and high patient throughput.

Madinah provides an important setting for studying these relationships because substantial numbers of pilgrims and visitors receive healthcare services in the city during Ramadan. Saudi official statistics and health-sector reports document considerable pilgrimage activity and healthcare-service provision to pilgrims and visitors in Makkah and Madinah [9,10]. These data provide contextual evidence of healthcare-service demand during Ramadan; however, direct imaging workload data comparing Ramadan with other periods were not available for the present study. Therefore, Ramadan is considered the temporal and occupational context of this study rather than a demonstrated cause of increased imaging workload or musculoskeletal symptoms.

International and Saudi studies have reported high levels of WRMSDs among radiologists, radiology technologists and ultrasound technologists [11,12,13,14,15,16,17,18,19,20]. However, most existing work has focused on overall prevalence, anatomical distribution or general occupational risk factors.

To our knowledge, no previous study has investigated WRMSDs during Ramadan. Ramadan is characterized by the physiological and behavioral changes mentioned above, which may influence musculoskeletal symptoms. However, because occupational exposures are well-established and modifiable risk factors for WRMSDs, this study aimed to estimate the seven-day prevalence of WRMSDs and examine associations between selected demographic and occupational characteristics and pain intensity among radiology and ultrasound technologists working during Ramadan in hospitals in Madinah, Saudi Arabia.

## 2. Materials and Methods

### 2.1. Professional Terminology

In Saudi Arabia, the professional title radiology technologist corresponds to practitioners commonly referred to internationally as radiographers. Ultrasound technologist corresponds to sonographers. In addition, WRMSDs in this study refer to self-reported musculoskeletal complaints assessed using the NMQ in an occupational population, not clinically confirmed occupational diagnoses.

### 2.2. Sample Size

The minimum sample size was calculated using the single-population proportion formula for cross-sectional studies. An expected WRMSD prevalence of 75% was used because published evidence indicates a high prevalence of WRMSDs among imaging professionals, including a pooled prevalence of 75.8% among sonographers and similarly high estimates in Saudi imaging populations [12,19,20]. Assuming a 95% confidence level and a 5% margin of error, the initial required sample size was 289 participants. After applying finite population correction for an estimated eligible population of approximately 750 radiology and ultrasound technologists in Madinah, the minimum required sample size was 209 participants. The final analytical sample included 237 participants, exceeding the minimum required sample size of 209. The sample-size calculation was specifically designed for estimation of WRMSD prevalence and was not intended as a formal power calculation for individual subgroup comparisons or multivariable regression coefficients.

### 2.3. Study Design and Setting

A cross-sectional online survey was conducted in Madinah, Saudi Arabia, during Ramadan 1447/2026. Data were collected between the 8th and 15th days of Ramadan (25 February to 4 March 2026). This period was selected to correspond with the seven-day symptom-recall period and to ensure that participants had experienced Ramadan working conditions before completing the questionnaire. Ramadan is considered the study period and occupational context; the study did not include a pre-Ramadan or non-Ramadan comparison period.

### 2.4. Ethical Approval

Ethical approval was granted by the Local Medical Research Ethics Committee (TU-AMS-26-01-006). Electronic informed consent was obtained before participants accessed the survey questions. No direct personal identifiers were collected.

### 2.5. Survey Instrument and Pilot Testing

The survey was created using Microsoft Forms (Microsoft Corporation, Redmond, WA, USA). The English-language questionnaire included the Nordic Musculoskeletal Questionnaire (NMQ) [21], a 0–10 numeric pain rating scale (NPRS) [22], and demographic and occupational questions. Both instruments have been widely used in peer-reviewed occupational health research, including studies involving healthcare professionals and Saudi populations. In the present study, the NMQ was used to identify self-reported musculoskeletal complaints across predefined anatomical regions within an occupational population. The term WRMSDs is used in accordance with terminology commonly applied in comparable occupational studies using the NMQ; however, the questionnaire does not constitute a clinical diagnosis or establish occupational causation for each reported symptom.

The exact questionnaire administered to participants is provided as Supplementary Material. The questionnaire was reviewed by two experts in radiology and healthcare and was subsequently piloted with 16 radiology and ultrasound technologists to assess clarity and usability. The pilot was not intended as a psychometric validation study. Pilot participants were excluded from the final analysis.

### 2.6. Participants

Participants were eligible if they were full-time radiology or ultrasound technologists working in radiology departments in Madinah hospitals during Ramadan, had at least one year of professional experience, and were actively working in at least one imaging modality during the study period.

Participants were excluded if they were on leave during the study period, were not actively working in an imaging modality, or self-reported a pre-existing chronic musculoskeletal disorder according to the eligibility question in the survey. The latter criterion was based on self-report and was not independently verified through clinical records. Participation was voluntary, and no incentives were offered.

### 2.7. Recruitment and Survey Distribution

Participants were recruited using online convenience and snowball sampling. The questionnaire was distributed through professional email networks and medical-radiology communities on Telegram, WhatsApp, and X, with personal professional contacts also supporting snowball distribution. The survey opened on 8 Ramadan 1447 (25 February 2026) and closed on 15 Ramadan 1447 (4 March 2026).

Because recruitment occurred through overlapping online professional networks rather than through a predefined hospital-based sampling frame, a formal response rate could not be calculated. Hospital-specific eligible population counts and facility-level recruitment information were not collected.

### 2.8. Statistical Analysis

Data were analysed using Stata/MP version 17.0 (StataCorp, College Station, TX, USA). Continuous variables were summarised as mean ± standard deviation (SD), and categorical variables as frequencies and percentages. Pain intensity, measured using the 0–10 NPRS, was modelled as a continuous outcome.

The distribution of continuous variables was assessed using graphical methods and normality tests. Age, BMI and pain intensity showed mild deviations from normality; however, parametric analyses were retained because of the sample size and the robustness of these methods to mild non-normality. Bivariate associations were examined using independent-samples *t*-tests for dichotomous variables, one-way analysis of variance for categorical variables with more than two groups, and Pearson correlation coefficients for continuous variables. Levene’s test was used to assess homogeneity of variance. Where the homogeneity-of-variance assumption was violated, Welch’s ANOVA was used, with non-parametric sensitivity analyses where appropriate.

Variables identified from the bivariate analyses together with clinically and occupationally relevant variables were considered for inclusion in the multivariable linear regression model. Robust standard errors were used. Multicollinearity was assessed using variance inflation factors (VIFs). Years of professional experience were excluded from the final model because of substantial collinearity with age. Regression coefficients are reported as unstandardized B values with 95% confidence intervals. Statistical significance was set at *p* < 0.05.

## 3. Results

### 3.1. Participant Characteristics

A total of 237 radiology and ultrasound technologists were included in the analysis. The mean age was 35.3 ± 7.4 years, and the mean BMI was 27.4 ± 3.8 kg/m^2^. Females constituted 52.7% of the sample, while males accounted for 47.3%. Most participants were radiology technologists (82.3%), and 17.7% were ultrasound technologists.

Approximately one-third of participants had at least 15 years of professional experience (30.8%). Most worked 8–9 h per shift (79.3%), managed 11–20 patients per shift (41.4%), and reported no night-shift duties (32.5%). Mean pain intensity was 5.55 ± 2.28 on the 0–10 numeric pain rating scale (Table 1).

### 3.2. Seven-Day Prevalence of Musculoskeletal Symptoms

**Figure 1:**
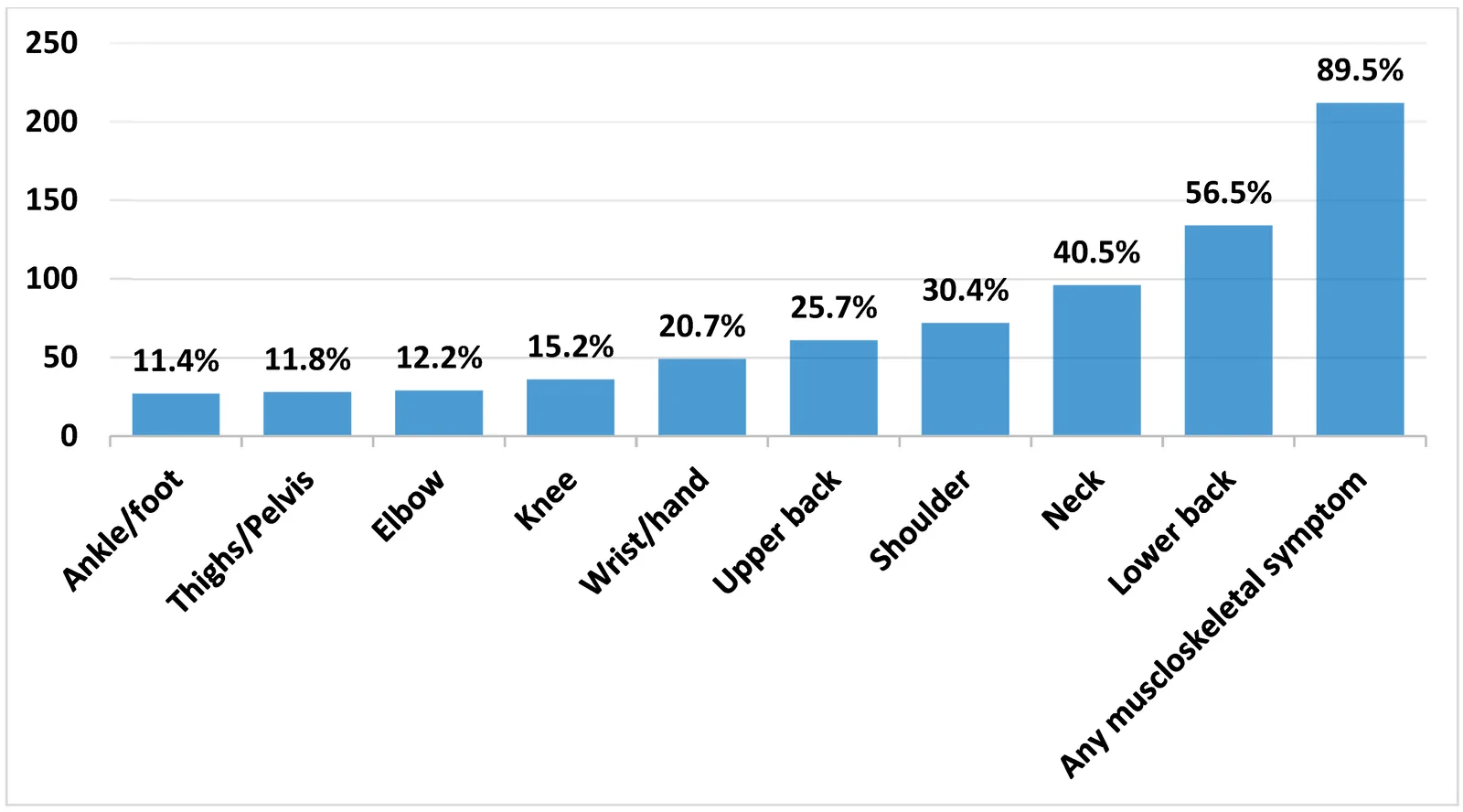
Seven-day prevalence of musculoskeletal symptoms by anatomical region. Bars show the number of participants reporting symptoms in each anatomical region, with the corresponding prevalence percentages (%) displayed above the bars. “Any musculoskeletal symptom” represents the aggregate number and percentage of participants reporting symptoms in at least one anatomical region and is not an individual anatomical region.

Overall, 212 participants (89.5%) reported musculoskeletal symptoms in at least one anatomical region during the preceding 7 days. The lower back was the most affected region (56.5%), followed by the neck (40.5%) and shoulders (30.4%) (Table 2 and Figure 1).

Participants could report symptoms in more than one anatomical region. The variable “any WRMSD” was a derived aggregate measure indicating symptoms in at least one assessed anatomical region and was not a separate anatomical item in the NMQ.

### 3.3. Factors Associated with Pain Intensity

**Figure 2:**
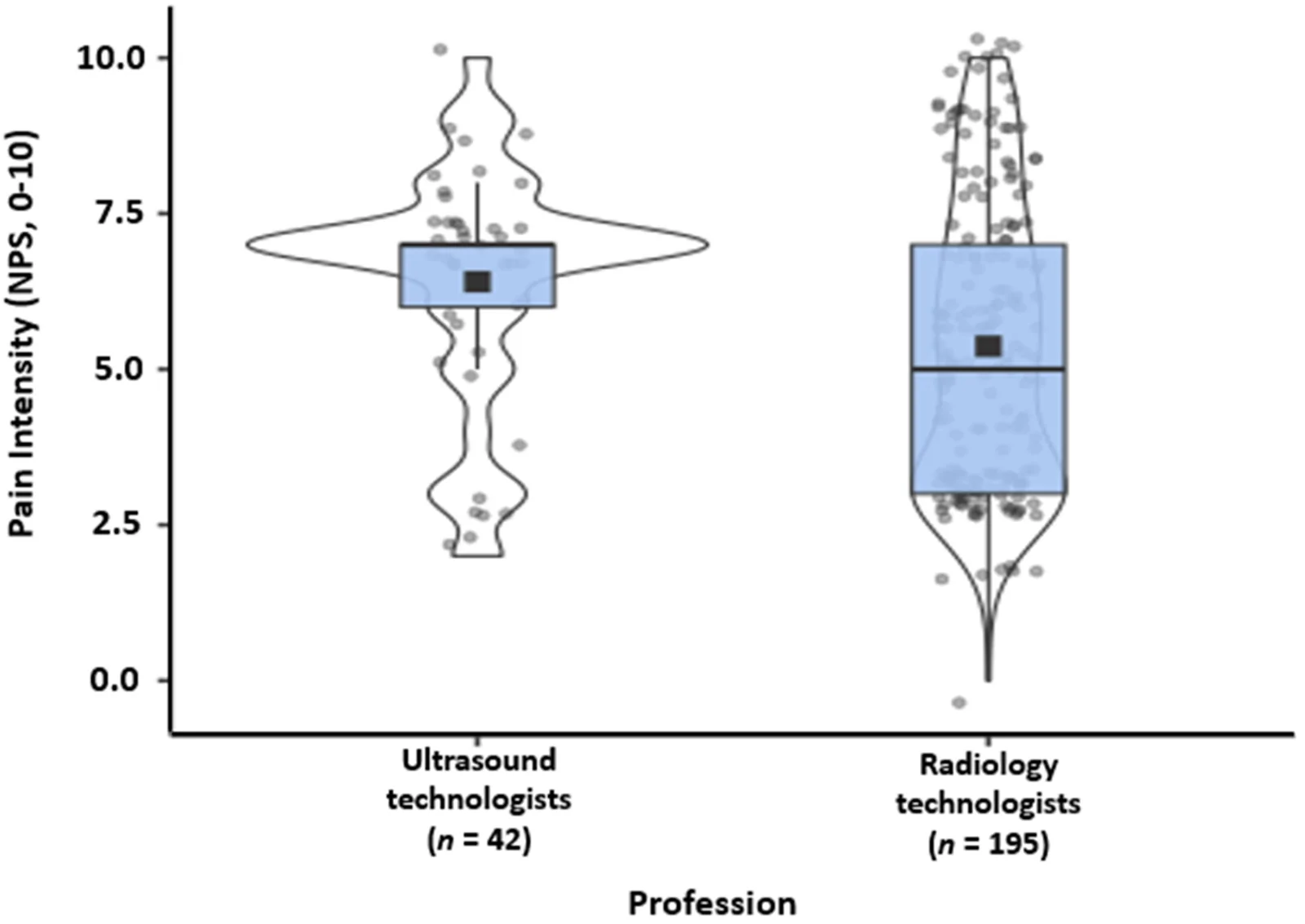
Distribution of pain intensity scores (0–10 Numeric Pain Rating Scale [NPRS]) according to profession among radiology and ultrasound technologists. Violin plots illustrate the distribution of pain scores, box plots display the median and interquartile range (IQR), black squares denote the mean, and dots represent individual participants.

**Figure 3:**
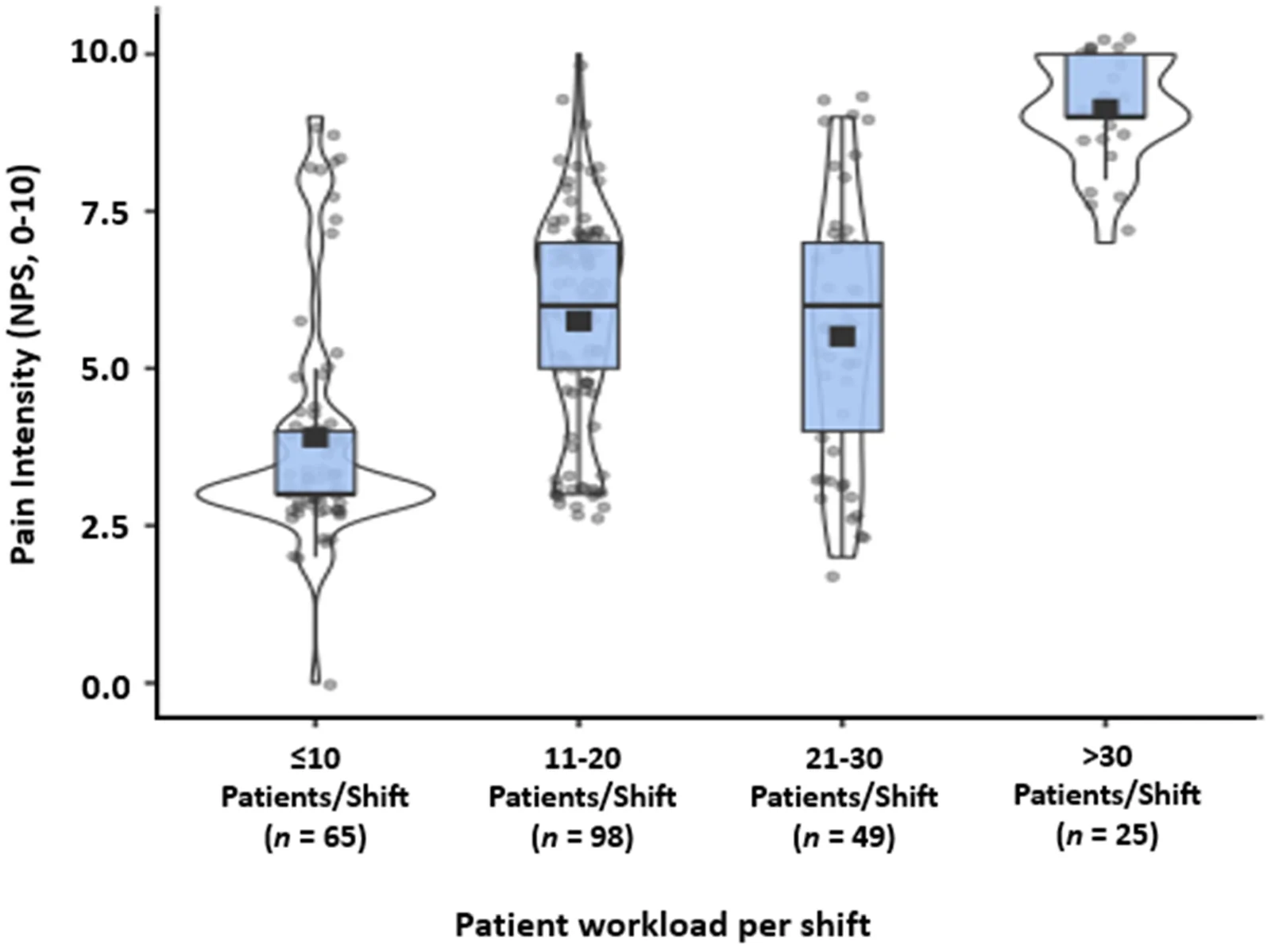
Distribution of pain intensity scores (0–10 Numeric Pain Rating Scale [NPRS]) according to patient workload per shift among radiology and ultrasound technologists. Violin plots illustrate the distribution of pain scores, box plots display the median and interquartile range (IQR), black squares denote the mean, and dots represent individual participants.

**Figure 4:**
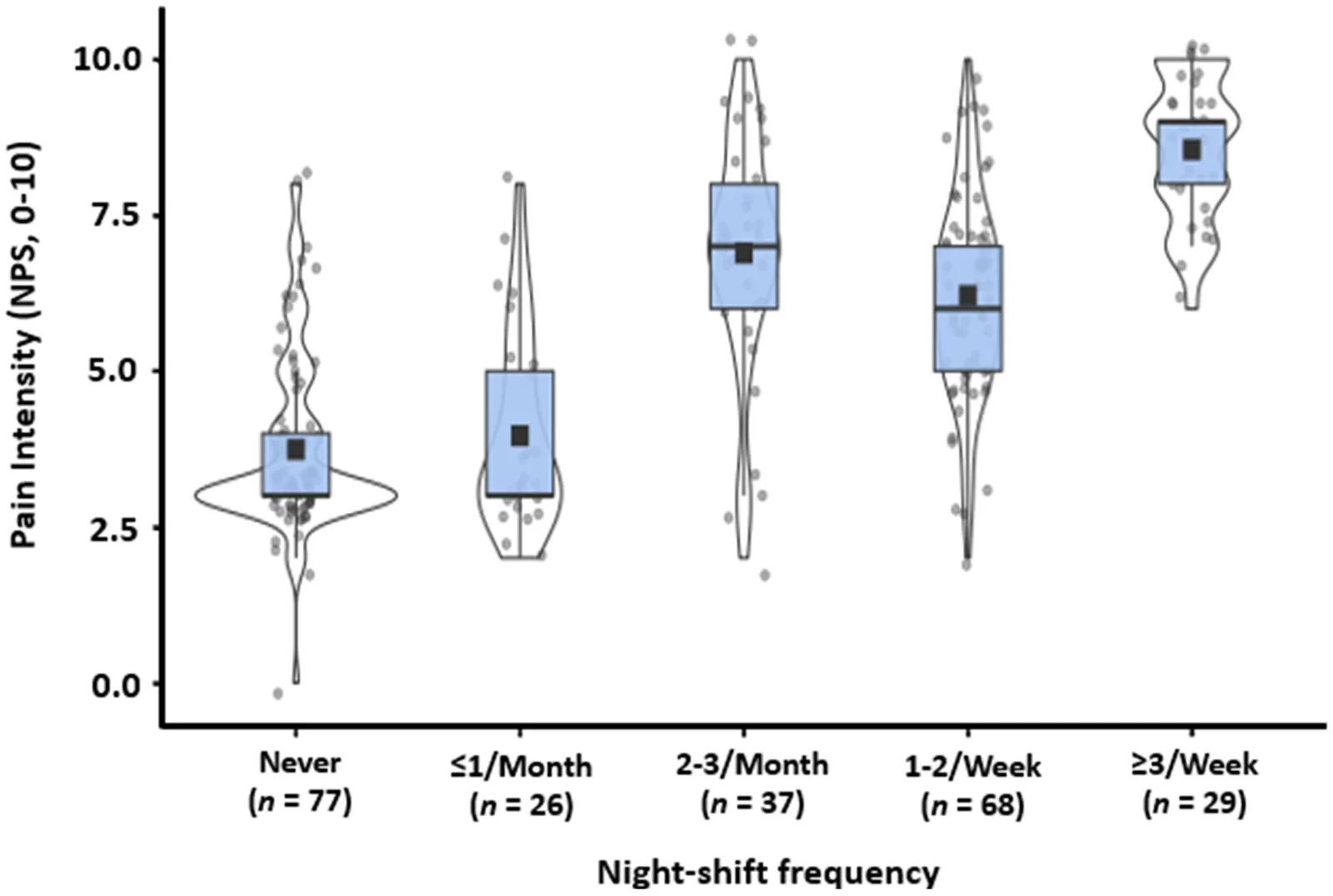
Distribution of pain intensity scores (0–10 Numeric Pain Rating Scale [NPRS]) according to night-shift frequency among radiology and ultrasound technologists. Violin plots illustrate the distribution of pain scores, box plots display the median and interquartile range (IQR), black squares denote the mean, and dots represent individual participants.

Pain intensity was weakly but significantly correlated with age (r = 0.216, *p* < 0.001) and BMI (r = 0.253, *p* < 0.001). Mean pain intensity also differed by sex and profession. Males reported higher pain scores than females. Ultrasound technologists reported higher pain intensity than radiology technologists (6.40 ± 1.91 vs. 5.37 ± 2.32; t = 2.70, *p* = 0.007) (Figure 2). Pain intensity also differed across patient-load categories (Figure 3), although the pattern was not monotonic. The highest adjusted pain intensity was observed among participants managing more than 30 patients per shift. Pain intensity also varied according to night-shift frequency (Figure 4), with higher adjusted pain scores among participants working 2–3 nights per month, 1–2 nights per week, and ≥3 nights per week compared with those who never worked night-shifts. The largest adjusted association was observed in the ≥3 nights-per-week category (Table 3).

### 3.4. Multivariable Linear Regression

A multivariable linear regression model with robust standard errors was constructed to examine factors associated with pain intensity after adjustment for the variables included in the model. Years of professional experience was excluded from the final model because of substantial collinearity with age. The final model was statistically significant (F(9, 227) = 72.49, *p* < 0.001) and explained 65.2% of the variance in pain intensity (R^2^ = 0.652).

In the adjusted model, increasing age was associated with higher pain intensity (B = 0.035, 95% CI 0.007 to 0.063, *p* = 0.015). Compared with ultrasound technologists, radiology technologists reported lower pain scores (B = −0.77, 95% CI −1.25 to −0.28, *p* = 0.002), indicating higher age-adjusted pain intensity among ultrasound technologists (Table 4).

Patient workload was associated with pain intensity, although the pattern was not fully linear across all categories. Compared with participants managing ≤10 patients per shift, those managing 11–20 patients per shift had higher pain scores (B = 0.71, 95% CI 0.10 to 1.33, *p* = 0.024), while those managing 21–30 patients did not differ significantly (B = 0.08, 95% CI −0.68 to 0.84, *p* = 0.835). Participants managing more than 30 patients per shift had substantially higher pain intensity (B = 2.75, 95% CI 1.86 to 3.64, *p* < 0.001) (Table 4).

Night-shift frequency showed the strongest adjusted association with pain intensity. Compared with participants who never worked night-shifts, those working 2–3 nights per month (B = 2.20, 95% CI 1.48 to 2.92, *p* < 0.001), 1–2 nights per week (B = 1.99, 95% CI 1.27 to 2.71, *p* < 0.001) and at least three nights per week (B = 3.27, 95% CI 2.43 to 4.10, *p* < 0.001) reported higher pain scores. The highest pain intensity was observed in the most frequent night-shift category presented in Table 4. BMI, sex, years of experience and shift duration were not independently associated with pain intensity after adjustment. Multicollinearity was assessed using variance inflation factors (VIFs), and no problematic multicollinearity was identified in the final model.

## 4. Discussion

This study found a high seven-day prevalence of WRMSDs among radiology and ultrasound technologists working during Ramadan in Madinah. The lower back, neck and shoulder were the most frequently affected anatomical regions. These findings are consistent with previous studies among radiographers, sonographers, and other healthcare professionals reporting substantial musculoskeletal burden in these regions [23,24,25]. The principal occupational findings of the present study were the associations of pain intensity with profession, patient workload, and night-shift frequency after adjustment for the variables included in the model.

The prevalence of lower back pain (56.5%) observed in this study is comparable to estimates reported among healthcare professionals in Turkey [26], Italy [27], Saudi Arabia [28], and China [29]. The important contribution of this study is not simply that WRMSDs are common, which is already well-established, but that pain intensity was strongly associated with night-shift frequency and very high patient workload during Ramadan.

Ultrasound technologists reported higher pain intensity than radiology technologists after adjustment. This is plausible and consistent with systematic reviews and international studies showing a high WRMSD burden among sonographers [20,30]. Ultrasound work often requires constrained postures, sustained shoulder abduction, static trunk positions, repetitive scanning movements and prolonged transducer grip. Saudi and international studies have reported similar profession-specific risks among radiology and ultrasound practitioners [14,15,16,17,18,19,31,32,33,34,35]. For example, similar findings have been reported in Saudi Arabia [11,12,15,16,17,18], China [31,32,36], Pakistan [33], and the United States [30]. This finding suggests that ultrasound technologists may require targeted ergonomic assessment rather than being grouped with radiology technologists as a single occupational category [34].

Night-shift frequency showed the largest adjusted association with pain intensity among the occupational variables included in the model. This finding matters because night work during Ramadan may combine occupational fatigue with fasting-related disruption of sleep timing and recovery. Previous studies have linked sleep disturbance and occupational fatigue with musculoskeletal symptoms [35,37]. During Ramadan, fasting schedules and altered meal timing may contribute to disruptions in sleep–wake patterns and circadian rhythms [38]. Ramadan-specific evidence also suggests that disrupted sleep–wake cycles may influence metabolic responses to physical activity during fasting [39]. In healthcare settings characterized by prolonged standing, patient handling, and repetitive tasks, these physiological changes may exacerbate musculoskeletal discomfort and reduce workers’ capacity to cope with high physical demands. These factors may impair recovery from occupational loading and amplify pain perception. However, because sleep duration, hydration and fatigue were not measured directly in this study, this interpretation should be treated as plausible rather than proven. The study assessed night-shift frequency during Ramadan but did not collect detailed information on exact night-shift hours, rotating or consecutive schedules, rest intervals, opportunities for sleep during shifts, or recovery days. These factors may influence musculoskeletal recovery and should be examined in future studies.

Patient workload was also associated with pain intensity; however, the pattern did not support a simple dose–response relationship. Participants managing more than 30 patients per shift reported substantially higher adjusted pain scores than those managing ≤10 patients, whereas the 21–30 patient category did not differ significantly from the reference group. Therefore, the clearest finding concerns the highest patient-load category rather than a progressive increase across all workload categories. Patient count should also be interpreted as an approximate measure of workload rather than a direct measure of ergonomic exposure. Examination duration, procedure complexity, patient dependency, patient-transfer requirements, and physical demands differ between radiography and ultrasound procedures and were not measured directly in this study.

The Madinah setting may magnify occupational demands on healthcare workers during Ramadan. As one of Islam’s holiest cities, Madinah receives large numbers of Umrah pilgrims during the fasting month [40]. Recent Saudi government reports have documented millions of Umrah performers visiting Makkah and Madinah during Ramadan and throughout the Umrah season [10], which may contribute to increased demand for healthcare and support services. This temporary surge in service utilization may contribute to higher workloads among healthcare professionals. Similar relationships between workload and WRMSDs have been reported among radiology technologists [41,42], and other healthcare workers [29,43]. However, the present study did not directly compare imaging workload during Ramadan with non-Ramadan periods. Consequently, the observed workload and musculoskeletal findings cannot be attributed specifically to Ramadan.

Age remained associated with pain intensity after adjustment for the variables included in the model. This may reflect cumulative occupational exposure, age-related changes in tissue resilience and slower recovery from mechanical loading. Because years of experience were collinear with age and were not retained in the final model, the age coefficient should not be interpreted as a purely biological effect. It is more likely to represent a mixture of ageing and accumulated work exposure, consistent with previous evidence from healthcare workers and other physically demanding clinical roles. This interpretation is consistent with healthcare-worker studies reporting associations between musculoskeletal symptoms and demographic or employment-related factors, although the specific contribution of age cannot be separated fully from accumulated occupational exposure in this cross-sectional analysis [44,45].

The findings suggest that workload distribution, night-shift scheduling, and ergonomic practices may be relevant targets for occupational health evaluation in imaging departments. Particular attention may be warranted for ultrasound technologists and staff exposed to very high patient-loads or frequent night-shifts. Ergonomic assessment, appropriate recovery periods, and workload redistribution could be considered in future intervention studies. Because the present study was cross-sectional, the effectiveness of these strategies cannot be determined from the current findings.

This study has several strengths. Data were collected during Ramadan itself rather than retrospectively after the month had ended. The study also examined both symptom prevalence and pain intensity, which provides a more informative assessment. The use of recognised instruments and the inclusion of both radiology and ultrasound technologists make the findings helpful for occupational health planning in imaging departments.

### 4.1. Study Limitations

Several limitations should be considered when interpreting the findings. First, the cross-sectional design prevents determination of temporality or causality and allows the possibility of reverse causality; for example, workers experiencing pain may have had their workload or shift patterns modified. Second, exposures and outcomes were self-reported, which may introduce recall, reporting, and common-method bias. Third, recruitment used online convenience and snowball sampling, a formal response rate could not be calculated and volunteer or selection bias is possible.

The study was conducted during Ramadan without a pre-Ramadan or non-Ramadan comparison period; therefore, the observed prevalence and associations cannot be attributed specifically to Ramadan fasting or Ramadan-related healthcare demand. Individual fasting status, sleep duration and quality, hydration, fatigue, psychosocial stress, depression, burnout, analgesic medication use, physical activity, previous injury, work breaks, recovery days, and detailed ergonomic exposures were not measured. Residual confounding from these and other unmeasured factors is therefore possible.

Patient count was used as an indicator of workload but did not capture procedure duration, complexity, patient dependency, or modality-specific biomechanical demands. Detailed night-shift organization, including exact hours, rotation patterns, rest intervals, and recovery periods, was also not assessed. In addition, ultrasound technologists represented a smaller proportion of the sample than radiology technologists, and some occupational subgroups were relatively small.

Finally, participants reporting pre-existing chronic musculoskeletal disorders were excluded. Although this was intended to reduce the influence of longstanding conditions on pain assessment, it may have excluded workers whose chronic conditions were partly related to previous occupational exposure and could therefore have underestimated the overall musculoskeletal burden. The findings should consequently be generalized cautiously beyond the study population and setting.

### 4.2. Future Directions

Future longitudinal studies should compare Ramadan and non-Ramadan periods to better characterize temporal changes in musculoskeletal symptoms. Studies should incorporate objective workload and ergonomic assessments, modality-specific exposure measures, sleep and fatigue indicators, hydration, psychosocial factors, analgesic use, recovery days, and detailed shift-scheduling characteristics. Prospective and intervention studies are also needed to determine whether modifications in workload distribution, night-shift scheduling, ergonomic practices, and recovery periods reduce pain intensity or the persistence of musculoskeletal symptoms among radiology and ultrasound technologists.

## 5. Conclusions

This study aimed to estimate the seven-day prevalence of WRMSDs and examine occupational factors associated with pain intensity among radiology and ultrasound technologists working during Ramadan in Madinah. WRMSDs were highly prevalent, with the lower back, neck, and shoulder being the most frequently affected anatomical regions. Ultrasound technologists reported higher adjusted pain intensity than radiology technologists, while frequent night-shift work and the highest patient-load category showed the largest occupational associations with pain intensity.

These findings identify workload distribution, night-shift scheduling, and profession-specific ergonomic exposure as potential areas for occupational health evaluation. However, because the study was cross-sectional and did not include a non-Ramadan comparison period, the findings represent associations observed during Ramadan and should not be interpreted as evidence that Ramadan itself or the measured occupational factors caused the reported symptoms. Prospective studies are required to determine whether modifying workload, scheduling, and ergonomic conditions reduces musculoskeletal pain.

## Figures and Tables

**Table 1:** Sociodemographic and occupational characteristics of participants.

Factors	Variable	Mean ± SD	Frequency (n)	Percentage (%)
Demographic	Age, years	35.34 ± 7.36	*-*	*-*
	BMI, kg/m^2^	27.40 ± 3.76	*-*	*-*
Pain scale	Pain intensity, 0–10 numeric pain rating scale	5.55 ± 2.28	*-*	*-*
Profession	Ultrasound technologist	-	42	17.72%
	Radiology technologist	-	195	82.28%
Sex	Female	-	125	52.74%
	Male	-	112	47.26%
Years of professional experience	<5 years	-	51	21.52%
	5–9 years	-	42	17.72%
	10–14 years	-	71	29.96%
	≥15 years	-	73	30.80%
Shift duration during Ramadan	<6 h	-	4	1.69%
	6–7 h	-	26	10.97%
	8–9 h	-	188	79.32%
	≥10 h	-	19	8.02%
Patients per shift	≤10 patients	-	65	27.43%
	11–20 patients	-	98	41.35%
	21–30 patients	-	49	20.68%
	>30 patients	-	25	10.55%
Night-shift frequency	Never	-	77	32.49%
	≤1 night per month	-	26	10.97%
	2–3 night-shifts per month	-	37	15.61%
	1–2 night-shifts per week	-	68	28.69%
	≥3 night-shifts per week	-	29	12.24%

**Table 2:** Distribution of seven-day prevalence of work-related musculoskeletal symptoms by anatomical region.

Body Region	Yes *n* (%)	No *n* (%)	Total
Neck	96 (40.51%)	141 (59.49%)	237
Shoulder	72 (30.38%)	165 (69.62%)	237
Upper back	61 (25.74%)	176 (74.26%)	237
Elbow	29 (12.24%)	208 (87.76%)	237
Wrist/hand	49 (20.68%)	188 (79.32%)	237
Lower back	134 (56.54%)	103 (43.46%)	237
Thighs/Pelvis	28 (11.81%)	209 (88.19%)	237
Knee	36 (15.19%)	201 (84.81%)	237
Ankle/Foot	27 (11.39%)	210 (88.61%)	237
Any WRMSD (any region)	212 (89.45%)	25 (10.55%)	237

“Any WRMSD” represents a derived aggregate measure indicating symptoms in at least one anatomical region.

**Table 3:** Bivariate associations between study variables and pain intensity.

Variable	Test Statistic	*p*-Value
Age	r = 0.216	<0.001
BMI	r = 0.253	<0.001
Sex	t = −2.60	0.010
Profession	t = 2.70	0.007
Year of Experience	Welch F = 14.61	<0.001
Shift duration	Welch F = 24.10	<0.001
Patients per shift	Welch F = 52.82	<0.001
Night-shift frequency	Welch F = 70.46	<0.001

Males reported higher pain intensity than females.

**Table 4:** Multivariable linear regression model for factors associated with pain intensity.

Variable	Adjusted B	95% CI	*p*-Value
Age (years)	0.035	0.007, 0.063	0.015
Radiology technologist, ref: ultrasound technologist	−0.77	−1.25, −0.28	0.002
11–20 patients/shift, ref: ≤10 Patients/shift	0.71	0.10, 1.33	0.024
21–30 patients/shift, ref: ≤10 Patients/shift	0.08	−0.68, 0.84	0.835
>30 patients/shift, ref: ≤10 Patients/shift	2.75	1.86, 3.64	<0.001
≤1 night/month, ref: never worked night-shifts	−0.09	−0.77, 0.59	0.795
2–3 nights/month, ref: never worked night-shifts	2.20	1.48, 2.92	<0.001
1–2 nights/week, ref: never worked night-shifts	1.99	1.27, 2.71	<0.001
≥3 nights/week, ref: never worked night-shifts	3.27	2.43, 4.10	<0.001

Model statistics: R^2^ = 0.652, F(9, 227) = 72.49, *p* < 0.001. Reference categories were ultrasound technologist for profession, ≤10 patients per shift for workload and no night-shifts for night-shift frequency.

## Data Availability

The data presented in this study is available on request from the corresponding author. The data are not publicly available to protect participant privacy and confidentiality.

## Supplementary Materials

The following supporting information can be downloaded at https://www.mdpi.com/article/10.3390/healthcare14172718/s1, Study Questionnaire.

## Abbreviations

The following abbreviations are used in this manuscript:

WRMSDs	Work-related musculoskeletal disorders
BMI	Body mass index
SD	Standard deviation
NMQ	Nordic musculoskeletal questionnaire
NPRS	Numeric pain rating scale
IQR	Interquartile range
VIFs	Variance inflation factors
