# Supplementary material for: Work-Related Musculoskeletal Disorders Among Radiology and Ultrasound Technologists During Ramadan in Madinah, Saudi Arabia: A Cross-Sectional Study
Source: Healthcare (Basel). 2026 Aug 26;14(17):2718. doi: 10.3390/healthcare14172718 (PMC13565550; doi:10.3390/healthcare14172718)
Supplement: Supplementary file 1 [file healthcare-14-02718-s001.zip › healthcare-4453168-supplementary.pdf]

# Study Questionnaire

## Section A: Demographic & Occupational Information

1. Profession:
   - Radiological Technologist (Radiographer)
   - Ultrasound technologist (Sonographer)

2. Age (years): ….

3. Height (cm): ….

4. Weight (kg): ….

5. Sex:

   - Male
   - Female
6. Years of professional experience:
   - <5 years
   - 5-9 years
   - 10-14 years
   - ≥15 years
7. Average Shift Duration During Ramadan:
   - <6 hours
   - 6-7 hours
   - 8-9 hours
   - ≥10 hours
8. Average number of patients per shift during Ramadan:
   - ≤10 patients
   - 11–20 patients
   - 21–30 patients
   - >30 patients
9. How often do you work night shifts?
   - Never
   - ≤1 night shift per month
   - 2–3 night shifts per month
   - 1–2 night shifts per week
   - ≥3 night shifts per week

**Section B:**

**Numeric Pain Rating Scale (NPRS)**

10. On average, how would you rate your work-related musculoskeletal pain during last 7 days of Ramadan?

Please select a number from 0 to 10:

0 = no pain                                                                 10 = worst pain

| | | | | | | | | | | |
|---|---|---|---|---|---|---|---|---|---|---|
| 0 | 1 | 2 | 3 | 4 | 5 | 6 | 7 | 8 | 9 | 10 |

**Section C:**

**Nordic Musculoskeletal Questionnaire (NMQ)**

During the last 7 days of Ramadan, have you experienced trouble (ache, pain, discomfort, or numbness) in any of the following body regions?

11. Neck
    - Yes
    - No
12. Shoulders
    - Yes
    - No
13. Upper back
    - Yes
    - No
14. Elbows
    - Yes
    - No
15. Wrists/hands
    - Yes
    - No
16. Lower back
    - Yes
    - No
17. Hips/thighs
    - Yes
    - No
18. Knees
    - Yes
    - No
19. Ankles/feet
    - Yes
    - No
